# Research on Relevant Dimensions of Tourism Experience of Intangible Cultural Heritage Lantern Festival: Integrating Generic Learning Outcomes With the Technology Acceptance Model

**DOI:** 10.3389/fpsyg.2022.943277

**Published:** 2022-09-08

**Authors:** Xin-Zhu Li, Chun-Ching Chen, Xin Kang, Jian Kang

**Affiliations:** ^1^College of Design, National Taipei University of Technology, Taipei, Taiwan; ^2^School of Design, NingboTech University, Ningbo, China; ^3^Beijing United Gas Engineering & Technology Co., Ltd., Beijing, China

**Keywords:** augmented reality, technology acceptance, intangible cultural heritage tourism, Lantern Festival, generic learning outcomes

## Abstract

The lantern exhibition at the Lantern Festival is an important traditional festival in Taiwan. Visitors play an important role in the promotion and sustainable development of intangible cultural heritage (ICH). In recent years, the involvement of digital technology in traditional lantern design and shows has contributed to the protection, inheritance, and promotion of ICH, there remains less research on using augmented reality (AR) with ICH tourism. In this study, AR is used for ICH lantern exhibition to discuss the learning experience in lantern tourism and the relationship between technology acceptance and satisfaction from the perspective of visitors, as well as evaluate what AR has on improving visitors’ awareness and learning experience. Then, primary variables of the technology acceptance model (TAM) are combined with generic learning outcomes (GLOs) to integrate ICH, education, and technology to expand TAM, building a new model to study the ICH learning experience. A questionnaire and observation are used. Respondents are visitors participating in the AR lantern exhibition in Taiwan, which is designed by the author. There is a total of 200 questionnaires collected in the end. The result shows that knowledge and understanding (KU), attitudes and values (AV), activity, behavior, and progression (ABP), and enjoyment, inspiration, and creativity (EIC) from GLOs have a positive effect on technology acceptance and actual use (AU). Therefore, visitors are satisfied with innovative and interesting technology learning experiences, enhancing learning interest and results. Besides, the interaction of the AR system improves visitors’ learning motivation, which shows the combination of AR technology with ICH tourism helps improve cultural awareness.

## Introduction

The protection and sustainability of intangible cultural heritage (ICH) is a major issue in the world (Anderson and Gerbing, 1988; Gössling, 2018; Lu et al., 2019; Tian et al., 2020). Governments should be committed to raising the awareness of people, especially young people about ICH and its significance (UNESCO, 2003). Lanterns were included in China’s Intangible Cultural Heritage Safeguarding List (China Intangible Cultural Heritage Website, 2018), leading to more attention to the traditional Lantern Festivals. It is believed that cultural heritage tourism is the best way to make people know more about the ICH, which can not only attract people to travel (Deacon, 2004; Rodzi et al., 2013; Masoud et al., 2019) but also disseminate local culture during festivals (tom Dieck et al., 2018; Li, 2020). It has been proved that study drives travel (Crompton, 1979; Mannell and Iso-Ahola, 1987; Prentice et al., 1998), and people’s desire for knowledge to decide where they go and what they do there (Crompton and McKay, 1997). The concept that cultural heritage tourism is combined with education is proposed (Ritchie, 2003). Tourism is considered an important driving force for lifelong learning, but its relationship with education remains to be studied (Falk et al., 2012).

In recent years, digital technology has been widely used in protecting ICH, such as augmented reality (AR), virtual reality, somatosensory interaction, 3D animation, holographic projection, and so on. Visitors can experience the cultural significance of ICH through these digital technologies, disseminating and effectively promoting ICH. To some degree, TAM can be used to show whether people accept the way ICH is shown through digital technology (Davis, 1989). How digital technology affects visitors’ reactions, behaviors, and psychology is paid more attention (Han et al., 2021). So far, TAM has been developed, researched, and widely used in education. Academically, TAM has a dramatic effect on teachers and students (Tan and Hsu, 2018), while generic learning outcomes (GLOs) provide a framework for planning and evaluating visitors’ learning results after the visit.

There is a growing concern about the effect of digital technology on visitor experience across academic communities but they seldom study about how digital technology is applied to the ICH learning experience. Therefore, an AR system is developed for visitors based on the ICH lantern learning experience in this study. In this way, visitors can interact with lanterns, and discuss the learning experience and relationship between technology acceptance from their perspective.

## Theoretical Background

### Non-formal Learning in Intangible Cultural Heritage Tourism

As the traditional culture is being destroyed (Lu et al., 2019), the ecology of culture diversity loses balance. Education can effectively protect, inherit, and disseminate ICH (UNESCO, 2003) through formal learning at school and non-formal learning outside school, such as museums, exhibitions, and tourism. Learning ICH at school helps protect and inherit the traditional culture (Olanipekun et al., 2015; Abisuga-Oyekunle and Fillis, 2017). However, learning it in an informal environment also plays an important part (Falk et al., 2012), because public involvement contributes to the heritage of ICH (Yan and Chiou, 2021). Non-formal learning in the form of travel facilitates personal practical skill, knowledge, and intelligence, facilitating their intelligence, cross-cultural awareness, and professional development (Falk et al., 2012). In the past decade, cultural heritage tourism has played a more and more important part (Abuamoud et al., 2014). With the help of technology, visitors can know more about the environment, culture, religion, tradition, and history when traveling, and improve their learning experience through different forms of communication technology (Mortara et al., 2014).

**FIGURE 1:**
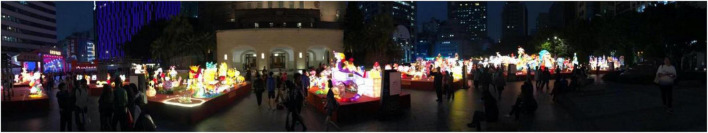
Taipei Lantern Festival in 2020.

The Lantern Festival is the day of the first full moon after the Spring Festival. Since 1990, Taiwan has held lantern exhibitions at the Lantern Festival every year, and 32 shows have been conducted up to now (Taiwan Tourism Bureau, 2021). At the Lantern Festival, various lantern competitions will be held across Taiwan, where different lantern works will be shown, aiming at inheriting lantern crafts. Recently, the lantern exhibition has been combined with technology and city tours, providing the public with more involvement and innovation (Li, 2020), and becoming a symbol of Taiwan. In 2020, for the first time, lantern exhibitions were held in two districts of Taipei. A total of 45 lantern stalls featuring a wide variety of lanterns attracted 2,834,939 visitors (Taipei City Government, 2020; see Figure 1). The lantern exhibition during the Lantern Festival is an ancient folk activity in the Chinese culture and has a long cultural tradition and strong cultural characteristics. The inheritance, protection, and transmission of ICH need the participation of all of the public. Media and education are important ways to spread ICH (Yang, 2017). To bring our excellent traditional culture into life, education is the first and the only way to protect the ICH.

In recent years, there have been discussions about how to measure the learning outcomes of non-formal learning. Learning outcomes refer to what the learning has achieved. It may be short term or long term, depending on personal understanding (Hooper-Greenhill et al., 2003). Bitgood and Loomis (1993) assumed that it was important to value visitors’ experiences. An increasing number of museums realize that experience was an important factor to affect experience value and visitors’ satisfaction (Rowley, 1999). As a result, they attach great importance to visitor-centered experience, which also drives the development and evolvement of measurement tools. These tools try to analyze the learning outcomes of visitors from different perspectives. Among them, the Learning Impact Research Project is one of the most representative. This project was entrusted to the Research Centre for Museums and Galleries, University of Leicester by the Museums, Libraries and Archives Council in 2001. This center proposed GLOs (Hooper-Greenhill et al., 2003; Hooper-Greenhill, 2004; Su, 2020), and hoped that museums, libraries, and galleries used this tool to measure the learning outcomes of non-formal learning.

Generic learning outcomes have become a framework for evaluating arts and culture, and consist of five parts: (1) knowledge and understanding (KU), (2) skills, (3) attitudes and values (AV), (4) enjoyment, inspiration, and creativity (EIC), and (5) activity, behavior, and progression (ABP), each of which plays an important part (Arts Council England, 2021). GLOs are designed as a tool for museums, libraries, and archives (Monaco and Moussouri, 2009, p. 318) to understand the learning outcome. Many scholars seek to use GLOs to measure the learning outcome and experience of visitors during the show (Leue et al., 2015; tom Dieck et al., 2018). Many studies about visitors’ learning experience focus more on specific learning environments, such as museums, sites, and zoos (Moscardo, 1996; Prentice et al., 1998; Falk and Dierking, 2000; Packer, 2006), or visitors, such as backpackers, cultural tourists, and the elderly (McKercher, 2002; Roberson, 2003; Pearce and Foster, 2007), but less on outdoor festival activities. About GLOs, learning outcomes are measured by asking learners (Hooper-Greenhill et al., 2003; Hooper-Greenhill, 2004). In this study, GLOs are combined with the technology acceptance model (TAM) to measure non-formal learning outcomes during lantern tours.

### Improving the Learning Experience of Intangible Cultural Heritage Tourism Through Digital Technology

Digital technology can improve visitors’ knowledge and skills about traditional lantern crafts during their tours. Many scholars have recently introduced digital technology to ICH shows, education, promotion, dissemination, and inheritance. For example, embodied and tangible interaction is always used for ICH learning and inheritance in the field of performing arts (dance and drama) (Grammatikopoulou et al., 2019). AR combines “real image” with “virtual computer image,” making users operate virtual 3D or 2D objects in a visible real-world environment. It has been widely used in education (Martin et al., 2011). AR experience raises visitors’ awareness of protecting cultural heritage (Han et al., 2021). It was found that students would pay more attention to learning in education with AR, thus improving their learning experience (Santos et al., 2014; González Vargas et al., 2020), which could further enhance knowledge acquisition (Moorhouse et al., 2019). AR enhances interaction, involvement, and individuation (Leue et al., 2015). The learning experience of visitors with handheld devices is different from that without handheld devices (Mikalef et al., 2012; Leue et al., 2015). The mobile makes museum apps and gallery apps accessible and improves visitors’ personalized and interactive experiences (Chang et al., 2015).

Digital technology can effectively improve the ICH learning experience. Some scholars have applied VR to social practices, rituals, and festival events. They transformed oral traditions into the real world, where visitors can involve themselves in virtual festivals or stories (Chang, 2015; Skovfoged et al., 2018). In this way, visitors’ satisfaction will be improved by combining ICH with the sightseeing tour, which will in turn make visitors more loyal to ICH tourism (Tian et al., 2020).

Garzón et al. (2019) sorted 64 articles about AR and education between 2010 and 2018 and found that AR had a dramatic impact on students’ learning outcomes, especially in engineering, arts, and humanities. Students are more willing to participate in learning in this learning environment with AR (Damala et al., 2008; Dunleavy et al., 2009). Therefore, AR is the potential to improve the tourism experience (Chung et al., 2015; Jung et al., 2015).

Digital technology is valued in user experience, of which the TAM receives the most attention. TAM is normally used to evaluate how users accept new technology and key factors affecting users (Davis, 1989). TAM is widely used in education (Elwood et al., 2006; Alenazy et al., 2019), and focuses on perceived usefulness (PU), perceived ease of use (PEOU), social influence, attitude toward using (ATU), and actual use (AU) (Davis, 1989). PU and PEOU decide behavioral intention and attitude. TAM is under development and expansion, such as TAM 2 (Venkatesh, 2000; Venkatesh and Davis, 2000) and TAM 3. Given TAM 2, a unified theory on technology acceptance and use is linked to e-commerce (Venkatesh et al., 2003). TAM 3 is used to evaluate what decides using intention and behavior in an e-learning environment (Venkatesh and Bala, 2008). As AR technology is widely used in tourism, visitors gained more knowledge. At the same time, there were many problems, including whether knowledge transfer is done, and how users accept the technology and the technical level of AR. Therefore, it is very important to know how users accept AR technology, and what decides and influences its acceptance.

### Sustainability of Intangible Cultural Heritage

The core of protecting ICH is enhancing its vitality and sustainability (Lenzerini, 2011). Combining ICH protection with tourism will generate a new protection form (Zandieh and Seifpour, 2020). In recent years, tourist attractions and travel agencies provide ICH experience tours to enhance the ability to integrate ICH across borders (Su et al., 2020), and respect for cultural diversity and human creativity, producing a sense of identity (UNESCO, 2003). ICH has been reshaped when adapting to different communities and populations and surroundings and interacting with its history. Studies revealed that learning has a positive effect on ICH sustainability (Gössling, 2018), such as maintaining cultural vitality and empowering people with knowledge to ensure ICH’s sustainability (Grant, 2012). Experience replaces goods and services (Pine et al., 1999), and becomes the main factor in driving economic development. Creative cultural experience will have a positive effect on visitors’ experience and well-being. Visitors who participate in the cultural experience feel like improving their cultural awareness (Ismagilova et al., 2015). Ritchie and Hudson (2009) indicated that one of the challenges travel planners face is tourist experience design. The sustainability of ICH is characterized by “tourism + education + technology” in the new era.

### Summary

Above all, the Lantern Festival is an important traditional festival in Chinese culture, and lanterns are one of the major elements. As human’s ICH, this kind of culture needs to be inherited and transmitted by way of education. The education, in turn, promotes learners to learn more through experiential learning, including physical experience, touching, and understanding, to inherit and transmit ICH.

Nowadays, technologies are involved in ICH in many studies, which are also focused on whether users learn and understand cultures during the interaction experience. Especially, studies about the inheritance of tangible cultural heritages are rarely based on ICH, especially the traditional festival ceremonies. Therefore, targeting the culture of the Lantern Festival, this study explores the application of digital technology in ICH. Regarding digital technology, this study combines AR technology with mobile devices because the Lantern Festival is an outdoor activity. Understanding users’ experience about new technology helps to gradually build the relationship between ICH and digital technology, and create a suitable relationship model.

This study discusses the relationship between interactive technology and the inheritance and transmission of ICH. Targeting the culture of the Lantern Festival, this study combines AR technology and mobile device. GLOs and TAM are used to measure users’ learning outcomes for ICH and during the AR Lantern Festival.

## Research Methodology and Hypotheses

Relevant literature review shows that AR can enhance visitors’ experience on ICH, but seldom studies are about learning outcomes of AR experience. This study explores the learning experience and relationship between AR acceptance with AR applying to ICH tourism from the perspective of visitors, as well as discusses the value and GLOs of AR lanterns and the relationship between technology acceptance. A questionnaire is an effective way to understand visitors’ experiences (tom Dieck et al., 2018; Othman et al., 2021). In this study, a lantern interaction system is designed to collect questionnaires and analyze study data after the ICH lantern exhibition at the Lantern Festival.

In this study, lantern AR interactive system (LARIS) is designed to get visitors’ experiences on exhibition. To collect and analyze relevant data, questionnaires are distributed to visitors after they experience ICH lantern exhibition. To be effective, questionnaires must be distributed after the exhibition (tom Dieck et al., 2018; Othman et al., 2021). Finally, a semi-structured interview is taken to get visitors’ deep insights about this experience. To supplement the quantitative data, qualitative analysis is made.

### Participants

This study was conducted during the lantern exhibition of the Lantern Festival in 2020. Researchers recruited 200 visitors, and all of them were willing to fill the questionnaires and signed informed consent forms. Participants were 18–60 year olds and were proficient in the traditional Chinese. Therefore, all materials are in traditional Chinese.

This study was conducted under the support of the Taiwan Ministry of Education. Involving no human experiment and the vulnerable in this study, only visitors were required to sign informed consents. The informed consent is divided into 12 parts: which are, respectively, study objectives; conditions and restrictions on participation; study methods and procedures; potential risks, occurrence, and remedies; efficiency and benefits for participants; contraindications and restrictions; shelf life, application, and confidentiality of research materials; compensation for damages; data processing technique after the end of the study; withdrawal from the study and how to process its data; commercial benefits derived from the study and its extended applications; rights; signature. The informed consents are in Chinese.

### Interaction Design

**FIGURE 2:**
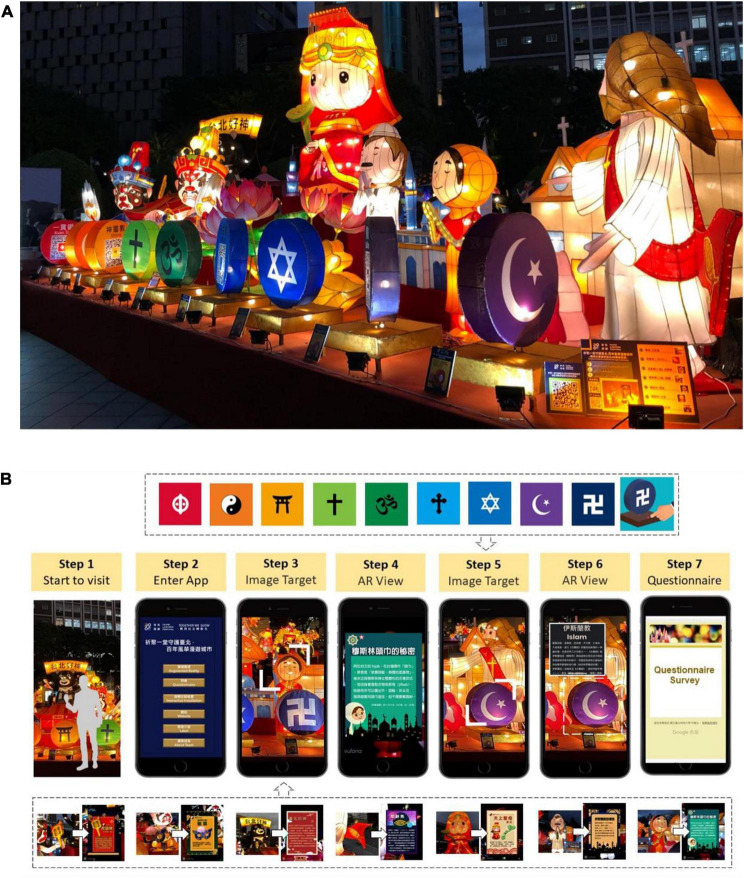
**(A)** Augmented reality lantern interactive design; **(B)** lantern AR interactive system (LARIS) use process.

During the 2020 lantern exhibitions at the Lantern Festival in Taiwan, LARIS was designed. With LARIS, visitors can get a description of the lantern they see. Its instructions are shown in Figure 2. Step 3 shows that you should align the lens with the image target (religious icon and religious lanterns), that is, a Muslim woman with a turban. Step 4 is what you get after scanning, where visitors know more about religious icons and religious lanterns (Figure 2).

### Questionnaire

The questionnaire is modified based on previous studies and experts’ advice to make it more efficient. It is divided into two parts: GLO items (Hung, 2014; Su et al., 2017; tom Dieck et al., 2018; Su, 2020; Li et al., 2021); and technology acceptance measurement items (Davis, 1989; Meng et al., 2018; Choi et al., 2019; Venkatesh, 2000; Venkatesh and Davis, 2000; Venkatesh et al., 2003). Its contents were checked by four experts, of whom two were museum planning curation experts, one was a HCI researcher and one was a lantern craftsman.

Likert scale was used, that is, strongly disagree, disagree, a little disagree, not sure, a little agree, agree, and strongly agree (Table 1). The study data were analyzed with SPSS 25.0 and AMOS 23.0, including confirmatory factor analysis (CFA) and structural equation modeling (SEM).

**TABLE 1:** Augmented reality lantern visit experiences questionnaires constructs and source.

Constructs	Number of items	References
**Part 1 generic learning outcomes items**		
Knowledge and understanding (KU)	4	Hung, 2014; tom Dieck et al., 2018; Su, 2020; Li et al., 2021
Attitudes and values (AV)	3	
Activity, behavior, and progression (ABP)	3	
Skills (S)	5	
Enjoyment, inspiration, and creativity (EIC)	4	
**Part 2 technology acceptance measurement items**		
Perceived ease of use (PEOU)	5	Davis, 1989; Esteban-Millat et al., 2018; Meng et al., 2018; Choi et al., 2019; Venkatesh and Davis, 2000
Perceived usefulness (PU)	3	
Attitude toward using (ATU)	3	
Behavioral intention to use (BIOU)	5	
Actual use (AU)	3	

### Hypothesis Development

Users will be affected by many factors when faced with new technology. These factors include PU, PEOU, behavioral intention to use (BIOU), ATU, and AU. PU refers to whether people think technologies are useful for him. PEOU refers to a specific system that can save people much energy (Davis, 1989; Venkatesh and Bala, 2008). BIOU is decided by ATU which refers to the general impression on technologies (Sun et al., 2008). PEOU will affect PU and is directly related to ATU, BIOU, and real system use (Esteban-Millat et al., 2018). PU is considered the most important factor to affect users’ technology acceptance.

**FIGURE 3:**
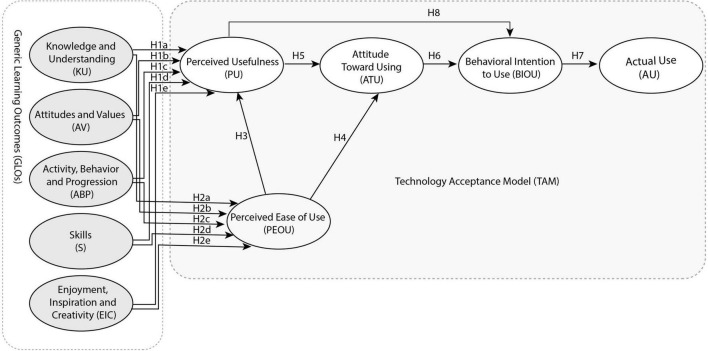
Research structure and hypothesis.

It is assumed that PU and PEOU are key driving factors in TAM variables. Besides, a TAM 2 framework was put forward, which revealed that cognitive tools affected PU, determining a person’s intention to use information systems finally (Venkatesh and Davis, 2000). With improvement, TAM 3 is built and shows the great differences between behavioral intention and using behavior (Venkatesh and Bala, 2008; Al-Gahtani, 2016). The relation between behavioral intention and user behavior has been built (Chuan-Chuan Lin and Lu, 2000). It is important to know what decides PU and its change with time and system use because they determine the AU. There are also external factors that affect PU and PEOU. In this article, GLOs of visiting AR lantern exhibitions are discussed as an external factor of the TAM framework. It also confirms the relevance of indicators above to various aspects of TAM and puts forward the hypotheses as given in Figure 3. A detailed explanation of the constructs and the justifications for the proposed hypotheses are highlighted in the following sections.

Davis (1989) pointed out that external variables enhanced the ability of TAM to predict people’s technology acceptance in the future, and might impact PU and PEOU. His idea was proved by many studies (Zare and Yazdanparast, 2013; Agudo-Peregrina et al., 2014) later. Bandura (1986) assumed that self-efficacy referred to a set of beliefs we hold about our ability to complete a particular task, evaluate self-competence and behavior, and self-influencing. The higher the self-efficacy is, the better people behave (Wang, 2020). For Wilhite (1990), academic self-efficacy refers to a kind of belief students hold about their ability to master learning behavior outcomes from the perspective of students’ learning. As is shown in many studies, academic self-efficacy will affect the PEOU and PU of new technology (Sánchez and Hueros, 2010; Fathema et al., 2015; Thongsri et al., 2020). Besides, GLOs are used to explore how AR technology affects visitors’ subjective learning outcomes (tom Dieck et al., 2018), which show that AR technology can enhance visitors’ understanding and value, improve their skills, increase their enjoyment and creativity, and affect their future behavior. Domain knowledge has a positive effect on PEOU (Thong et al., 2002), and perceived enjoyment influences PU and PEOU (Tao et al., 2019). Enjoyment will impact the user’s perceived value (Kim and Park, 2019). As is shown in previous studies, if users enjoy new technology, it will have a positive effect on PU and PEOU (Al-Aulamie et al., 2012). Therefore, subjective learning outcomes can be used to understand external variables of PEOU and PU is very important in explaining users’ technology acceptance. As a result, supposing that:

**Hypothesis 1 (H1):** Knowledge and understanding (KU) (H1a), skill (S) (H1b), enjoyment, inspiration, and creativity (EIC) (H1c), attitudes and values (AV) (H1d), and activity, behavior, and progression (ABP) (H1e) are positively correlated to perceived usefulness (PU).

**Hypothesis 2 (H2):** Knowledge and understanding (KU) (H2a), skill (S) (H2b), enjoyment, inspiration, and creativity (EIC) (H2c), attitudes and values (AV) (H2d), and activity, behavior, and progression (ABP) (H2e) are positively correlated to perceived ease of use (PEOU).

Perceived ease of use and PU are basic structures in TAM. Many studies confirm that PEOU has a dramatic impact on PU (Ayeh et al., 2013; Letchumanan and Muniandy, 2013; Abdullah et al., 2016; Hadi et al., 2022). As a result, supposing that:

**Hypothesis 3 (H3):** Perceived ease of use (PEOU) is directly and positively correlated with perceived usefulness (PU).

Whether this technology is feasible depends on users’ attitudes toward it (Fan, 2020). The relationship between PEOU and PU and ATU is based on the theory of reasoned action. According to this theory, PEOU and PU significantly affect ATU (Davis, 1989; Esteban-Millat et al., 2018). As a result, supposing that:

**Hypothesis 4 (H4):** Perceived ease of use (PEOU) is directly and positively correlated with attitude toward using (ATU).

**Hypothesis 5 (H5):** Perceived usefulness (PU) is directly and positively correlated with attitude toward using (ATU).

High TAU will affect users’ BIOU (Chung et al., 2015; Abdullah et al., 2016; Cheng and Yuen, 2018). If users have a good attitude toward the AR experience, they will have highly positive feelings, experiencing it again. Therefore, users’ high ATU for AR may affect its BIOU. As a result, supposing that:

**Hypothesis 6 (H6):** Attitude toward using (ATU) is positively correlated to behavioral intention to use (BIOU).

Behavioral intention to use refers to users’ intention to use the new system. The use of new technology depends on the users’ behavioral intention (Esteban-Millat et al., 2018). As is shown in previous studies, BIOU has a direct and significant effect on AU (Esteban-Millat et al., 2018; Salloum et al., 2019). As a result, supposing that:

**Hypothesis 7 (H7):** Behavioral intention to use (BIOU) is positively correlated to actual use (AU).

Davis (1989) said, if some kinds of technology could improve users’ working or learning performance, he would have a high BIOU. As a result, supposing that:

**Hypothesis 8 (H8):** Perceived usefulness (PU) is positively correlated to behavioral intention to use (BIOU).

### Data Collection and Analyses

A total of 202 visitors who participated in this study from the AR lantern exhibition were selected randomly between 8 and 16 February 2020. A total of 200 questionnaires were obtained finally except for 20 invalid questionnaires. The tourists were coded from V1 to V200. Then, SPSS 22.0 was used to make descriptive statistical analysis, and AMOS 23.0 was used to make SEM for fitting discussion and relationship among variables.

## Results

Sampling was done as the first test before a formal test with Cronbach alpha taking 0.956. If the result is greater than 0.7, the reliability is high (see Table 2).

**TABLE 2:** Reliability analysis.

	*N*	Cronbach’s alpha	
Generic learning outcomes	19	0.983	0.956
Technology acceptance measurement	19	0.983	

### Profile of Participants

As is seen in Table 3, the female visitors account for 67% (133), while the male visitors were 34% (67). Most of them were 31–40 year olds accounting for 33% (66). Visitors at the age of 21–30 years constituted 25% (50); visitors at the age of 41–50 years account for 25% (50); visitors at the age of 51–60 years were 12% (24) and visitors under 20 years old represented 5% (10). This exhibition successfully attracted many visitors who had never visited the lantern exhibition at the Lantern Festival.

**TABLE 3:** Summary of participants.

Demographic	Frequency	Percent	Cumulative percent
**Gender**			
Female	133	67	67%
Male	67	34	100%
Total	200	100	
**Age**			
Under 20	10	5	5%
21–30	50	25	30%
31–40	66	33	63%
41–50	50	25	88%
51–60	24	12	100%
Total	200	100	
**Education**			
Elementary school	14	7	7%
Junior high school	16	8	15%
Senior high school	58	29	44%
College	98	49	93%
Master/Ph.D.	14	7	100%
Total	200	100	
**Previous visit to the lantern exhibition in the Lantern Festival**			
Yes	54	27	27%
No	146	73	73%
Total	200	100	100%

### Measurement Model Verification

The measurement model of this study will be tested for validity, which is convergent validity and discriminant validity.

#### Measurement Model Verification

In this study, SEM, which is proposed by Anderson and Gerbing (1988), is used to evaluate and measure structural models. This method is divided into two steps. The first step aims to check the reliability and efficiency of CFA, and the second aims to measure path effects and their significance to the structural model. In the measurement model, maximum likelihood estimation (MLE) is used to evaluate factor loading, measurement reliability, convergent validity, and discriminant validity. Table 4 shows non-standard factor loading, standard factor loading, standard error, significance test, square multiple correlations, composite reliability (CR), and average variance extracted (AVE). Given all the questions of questionnaires, the standard factor loading was reasonable, ranging from 0.726 to 0.902, which shows the convergent validity. The CR is between 0.847 and 0.923, which is more than 0.7, a value recommended by Nunnally (1994). It shows the internal consistency of SEM. AVE value ranges from 0.604 to 0.821, more than 0.5 recommended by J. F. Hair, Anderson, Tatham, William, Fornell, and Larcker. It shows sufficient convergent validity (Fornell and Larcker, 1981; Anderson and Gerbing, 1988). Internal consistency is based on the relevance of different projects of the same test and is used to measure whether study items will generate similar scores.

**TABLE 4:** Confirmatory factor analysis.

Construct	Item	Significance of estimated parameters				Item reliability		Construct reliability	Convergence validity
		Unstd.	SE	*t*-Value	*P*-value	STD	SMC	CR	AVE
KU	KU1	1.000				0.726	0.527	0.853	0.592
	KU2	1.142	0.106	10.747	***	0.810	0.656		
	KU3	1.043	0.101	10.343	***	0.777	0.604		
	KU4	0.989	0.098	10.135	***	0.761	0.579		
AV	AV2	1.000				0.884	0.781	0.903	0.756
	AV3	0.887	0.055	16.245	***	0.868	0.753		
	AV4	0.932	0.059	15.903	***	0.857	0.734		
ABP	ABP3	1.000				0.885	0.783	0.900	0.750
	ABP4	0.972	0.057	16.991	***	0.882	0.778		
	ABP5	1.000	0.064	15.286	***	0.830	0.689		
Skill	Skill1	0.983				0.869	0.755	0.923	0.705
	Skill2	0.825	0.061	16.087	***	0.858	0.736		
	Skill3	0.875	0.059	13.925	***	0.790	0.624		
	Skill4	0.965	0.063	13.959	***	0.791	0.626		
	Skill5	1.000	0.057	17.012	***	0.885	0.783		
EIC	EIC2	1.054				0.818	0.669	0.896	0.682
	EIC3	1.114	0.079	13.359	***	0.821	0.674		
	EIC4	0.961	0.078	14.205	***	0.858	0.736		
	EIC5	1.000	0.074	13.018	***	0.806	0.650		
PEOU	PEOU1	0.889				0.795	0.632	0.915	0.682
	PEOU2	1.013	0.073	12.170	***	0.775	0.601		
	PEOU3	1.085	0.079	12.760	***	0.804	0.646		
	PEOU4	1.178	0.079	13.712	***	0.848	0.719		
	PEOU5	1.000	0.079	14.932	***	0.902	0.814		
PU	PU1	0.982				0.837	0.701	0.847	0.648
	PU3	0.883	0.073	13.513	***	0.804	0.646		
	PU4	1.000	0.069	12.745	***	0.773	0.598		
BIOU	BIOU1	0.979				0.819	0.671	0.915	0.684
	BIOU2	0.899	0.070	14.010	***	0.838	0.702		
	BIOU3	0.959	0.072	12.445	***	0.771	0.594		
	BIOU4	0.992	0.065	14.692	***	0.865	0.748		
	BIOU5	1.000	0.071	14.057	***	0.839	0.704		
ATU	ATU1	0.908				0.864	0.746	0.862	0.677
	ATU2	0.870	0.065	13.965	***	0.818	0.669		
	ATU4	1.000	0.066	13.109	***	0.784	0.615		
AU	AU2	1.095				0.757	0.573	0.849	0.652
	AU3	1.120	0.099	11.060	***	0.835	0.697		
	AU4	0.985	0.102	11.024	***	0.829	0.687		

*KU, knowledge and understanding; AV, attitudes and values; ABP, activity, behavior, and progression; S, skills; EIC, enjoyment, inspiration, and creativity; PEOU, perceived ease of use; PU, perceived usefulness; BIOU, behavioral intention to use; ATU, attitude toward using; AU, actual use.*

The symbol “***” indicates a SIG value less than 0.001, P-value test is “significant.”

#### Measurement Model Verification

Discriminant validity is used to judge the validity of different constructs and confirm whether the relevance of different constructs is different significantly. It can be achieved by comparing the square root of AVE and the relevance of different constructs (Fornell and Larcker, 1981; Segars and Grover, 1998). The coefficient proposed by Fornell and Larcker (1981) is more than 0.5, which shows the high internal consistency. Their high relevance means that things measured by them are the same. The standards above show that the AVE of each question of the questionnaire is more than 0.5, and the square root of AVE is more than the relevance coefficient of variables, which shows the better convergent validity and discriminant validity between various variables (Table 5).

**TABLE 5:** Discriminant validity for the measurement model.

	AVE	KU	AV	ABP	S	EIC	PEOU	PU	BIPU	ATU	AU
KU	0.592	**0.769**									
AV	0.756	0.576**	**0.869**								
ABP	0.750	0.568**	0.565**	**0.866**							
S	0.705	0.415**	0.372**	0.539**	**0.839**						
EIC	0.682	0.554**	0.565**	0.626**	0.496**	**0.825**					
PEOU	0.682	0.641**	0.615**	0.683**	0.506**	0.707**	**0.825**				
PU	0.648	0.670**	0.661**	0.679**	0.512**	0.728**	0.742**	**0.804**			
BIPU	0.684	0.513**	0.556**	0.630**	0.444**	0.535**	0.644**	0.682**	**0.827**		
ATU	0.677	0.567**	0.505**	0.583**	0.497**	0.626**	0.726**	0.709**	0.662**	**0.823**	
AU	0.652	0.273**	0.307**	0.362**	0.315**	0.285**	0.339**	0.353**	0.452**	0.305**	0.807

The diagonal elements in bold are the respective square root of the average variance extracted. **p < 0.01. KU, knowledge and understanding; AV, attitudes and values; ABP, activity; behavior and progression; S, skills; EIC, enjoyment, inspiration, and creativity; PEOU, perceived ease of use; PU, perceived usefulness; BIOU, behavioral intention to use; ATU, attitude toward using; AU, actual use.

#### Hypotheses Test

**Hypothesis 1:** In the analysis of the relation between GLOs and PU, PU is a dependent variable, and the following are independent variables aiming to determine the relative effect of various variables: knowledge and understanding (KU) (H1a), attitude, value, and enjoyment (AV) (H1b), actions and behavior change (ABP) (H1c), skills (S) (H1d), and enjoyment, inspiration, and creativity (EIC) (H1e). The results show that S (H1d) (*B* = 0.070, *t* = 1.274, *p* > 0.05) is not significantly affected, and therefore, H1d is assumed to be null without discussion. The independent variables below significantly affect PU: KU (H1a) (*B* = 0.193, *t* = 2.513, *p* < 0.05*), AV (H1b) (*B* = 0.174, *t* = 2.595, *p* < 0.01^**^), ABP (H1c) (*B* = 0.195, *t* = 2.463, *p* < 0.05*), and EIC (H1e) (*B* = 0.237, *t* = 2.883, *p* < 0.01^**^). The study confirmed that the hypotheses of H1a, H1b, H1c, and H1e are the case. After visiting lantern exhibitions with the AR system, visitors’ PU will be affected by KU, AV, ABP, and EIC, which will improve visitors’ PU when they visit lantern exhibitions with the AR system (Table 6).

**Hypothesis 3:** PEOU is taken as an independent variable and PU is taken as a dependent variable. The result shows that PEOU significantly affects PU (*B* = 0.219, *t* = 2.313, *p* < 0.01^**^) (Table 6), which indicates that H3 is the case where the PEOU of visitors will improve the PU.

**Hypothesis 2:** In the analysis of the relation between GLOs and PEOU, KU (H1a), AV (H1b), ABP (H1c), skills (H1d), and EIC (H1e) are taken as independent variables, and PEOU is taken as dependent variable to determine the relative effect of variables. The result showed that AV (H2b) (*B* = 0.114, *t* = 1.575, *p* > 0.05) and S (H2d) (*B* = 0.047, *t* = 0.749, *p* > 0.05) did not significantly affect PEOU. Therefore, H2b and H2d were not the cases, and there was no point in discussing. KU (H2a) (*B* = 0.226, *t* = 2.800, *p* < 0.01^**^), ABP (H2c) (*B* = 0.239, *t* = 2.866, *p* < 0.01^**^), and EIC (H2e) (*B* = 0.375, *t* = 4.552, *p* < 0.001^***^) significantly affected PEOU (Table 7), which confirmed that H2a, H2c, and H2e were the cases.

**TABLE 6:** Regression of H1 and H3.

Model	Unstandardized coefficients		Standardized coefficients	*t*-Value	*P*-value	SMC
	*B*	SE	Beta			
KU	0.212	0.084	0.193	2.513	0.012*	0.853
AV	0.121	0.046	0.174	2.595	0.009**	
ABP	0.128	0.052	0.195	2.463	0.014*	
S	0.057	0.045	0.070	1.274	0.203	
EIC	0.227	0.079	0.237	2.883	0.004**	
PEOU	0.212	0.092	0.219	2.313	0.021**	
a. Dependent variable: PU						

KU, knowledge and understanding; AV, attitudes and values; ABP, activity, behavior, and progression; S, skills; EIC, enjoyment, inspiration, and creativity; PEOU, perceived ease of use; PU, perceived usefulness. *Significant at p < 0.05, **significant at p < 0.01, ***significant at p < 0.001.

**TABLE 7:** Regression analysis for H2.

Model	Unstandardized coefficients		Standardized coefficients	*t*-Value	*P*-value	SMC
	*B*	SE	Beta			
KU	0.255	0.091	0.226	2.800	0.005**	0.733
AV	0.081	0.052	0.114	1.575	0.115	
ABP	0.162	0.057	0.239	2.866	0.004**	
S	0.040	0.050	0.047	0.794	0.427	
EIC	0.371	0.081	0.375	4.552	***	
a. Dependent variable: PEOU						

KU, knowledge and understanding; AV, attitudes and values; ABP, activity, behavior, and progression; S, skills; EIC, enjoyment, inspiration, and creativity; PEOU, perceived ease of use. *Significant at p < 0.05, **significant at p < 0.01, ***significant at p < 0.001.

After visiting lantern exhibitions with the AR system, the PEOU of visitors would be affected by KU, ABP, and EIC, which would improve visitors’ PEOU when they visit lantern exhibitions with the AR system.

**Hypothesis 4:** PEOU is taken as an independent variable, and ATU is a dependent variable. The result showed that PEOU significantly affected ATU (*B* = 0.445, *t* = 3.711, *p* < 0.001^***^). Therefore, H4 is the case, that is, the PEOU of visitors when visiting lantern exhibitions with the AR system will directly affect ATU (Table 8).

**Hypothesis 5:** PU is taken as an independent variable and ATU is taken as a dependent variable. The result showed that PU would significantly affect ATU (*B* = 0.440, *t* = 3.656, *p* < 0.001^***^). Therefore, H5 is the case, that is, the PU of visitors when visiting lantern exhibitions with the AR system will directly affect ATU (Table 8).

**Hypothesis 8:** PU is taken as the independent variable and BIOU is taken as the dependent variable. The result showed that PU will significantly affect BIOU (*B* = 0.528, *t* = 4.530, *p* < 0.001^***^). H8 is the case, that is, the PU of visitors when visiting lantern exhibitions with the AR system will directly affect BIOU (Table 9).

**Hypothesis 7:** BIOU is taken as the independent variable and AU is taken as the dependent variable. The results show BIOU will significantly affect AU (*B* = 0.508, *t* = 6.250, *p* < 0.001^***^). H7 is the case, that is, the BIOU of visitors when visiting lantern exhibitions with the AR system will directly affect AU (Table 10).

**TABLE 8:** Regression analysis for H4 and H5.

Model	Unstandardized coefficients		Standardized coefficients	*t*-Value	*P*-value	SMC
	*B*	SE	Beta			
PEOU	0.443	0.119	0.445	3.711	***	0.721
PU	0.452	0.124	0.440	3.656	***	
a. Dependent variable: ATU						

PEOU, perceived ease of use; PU, perceived usefulness; ATU, attitude toward using. *Significant at p < 0.05, **significant at p < 0.01, ***significant at p < 0.001.

**TABLE 9:** Regression analysis for H6.

Model	Unstandardized coefficients		Standardized coefficients	*t*-Value	*P*-value	SMC
	*B*	SE	Beta			
ATU	0.292	0.108	0.308	2.694	0.007**	0.639
PU	0.513	0.113	0.528	4.530	***	
a. Dependent variable: BIOU						

PU, perceived usefulness; BIOU, behavioral intention to use; ATU, attitude toward using. *Significant at p < 0.05, **significant at p < 0.01, ***significant at p < 0.001.

**TABLE 10:** Regression results for H7 and H8.

Model	Unstandardized coefficients		Standardized coefficients	*t*-Value	*P*-value	SMC
	*B*	SE	Beta			
BIOU	0.497	0.079	0.508	6.250	***	0.258
a. Dependent variable: AU						

BIOU, behavioral intention to use; AU, actual use. *Significant at p < 0.05, **significant at p < 0.01, ***significant at p < 0.001.

**FIGURE 4:**
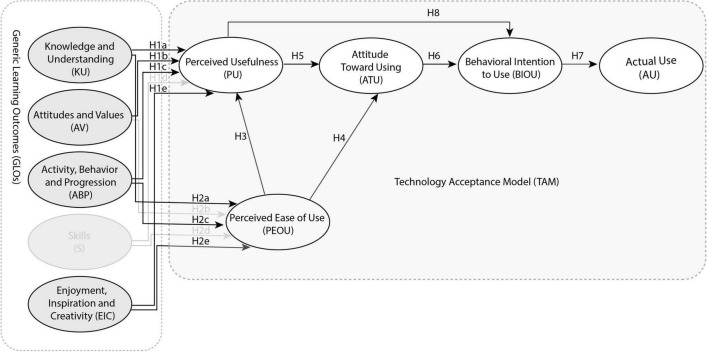
A summary of hypotheses with significant differences.

As is shown in Figure 4, H1a, H1b, H1c, H1e, H2a, H2c, H2e, H2, H3, H4, H5, H6, H7, and H8 is the case after statistical verification.

### Summary of Hypothesis Testing

In this study, 8 hypotheses were proposed, and are subdivided into 12 parts, of which 8 are supported and the rest are not supported (Table 11).

**TABLE 11:** Structural equation model analysis and hypotheses testing results.

Hypothesis		Relationship	Critical ratio or (*t*-value)	Supported	Result
H1	H1a	KU → PU	2.513	0.012*	Supported
	H1b	AV → PU	2.595	0.009**	Supported
	H1c	ABP → PU	2.463	0.014**	Supported
	H1d	S → PU	1.274	0.203	Not supported
	H1e	EIC → PU	2.883	0.004**	Supported
H2	H2a	KU → PEOU	2.800	0.005**	Supported
	H2b	AV → PEOU	1.575	0.115	Not supported
	H2c	ABP → PEOU	2.866	0.004**	Supported
	H2d	S → PEOU	0.794	0.427	Not supported
	H2e	EIC → PEOU	4.552	***	Supported
H3		PEOU → PU	2.313	0.021*	Supported
H4		PEOU → ATU	3.711	***	Supported
H5		PU → ATU	3.656	***	Supported
H6		ATU → BIOU	2.694	0.007**	Supported
H7		BIOU → AU	6.250	***	Supported
H8		PU → BIOU	4.530	***	Supported

KU, knowledge and understanding; AV, attitudes and values; ABP, activity, behavior, and progression; S, skills; EIC, enjoyment, inspiration, and creativity; PEOU, perceived ease of use; PU, perceived usefulness; BIOU, behavioral intention to use; ATU, attitude toward using; AU, actual use. *Significant at p < 0.05, **significant at p < 0.01, ***significant at p < 0.001.

### Structural Equation Modeling

#### Structural Equation Model Analysis

The overall model fit must be judged to verify the hypotheses. Schumacker and Lomax (2004) and Kline (2015) noted that model fit will be adversely affected with *p* less than 0.05 if it is analyzed with large quantities of samples. As a result, different methods should be used in a quantitative study to check model fit. In this study, generic models, which apply to verification methods, were implemented (Jackson et al., 2009). Based on these models, if Chi-square is divided into degree of freedom (df), the result is less than 3 ideally. Besides, other standards require stricter values in model fit verification, such as RMSEA value should be less than 0.08 (Hu and Bentler, 1999), the value of CFI standard should be more than 0.9. As is shown in Table 12, all model fitting standards which are tested conform to the recommended standard (Schumacker and Lomax, 2004).

**TABLE 12:** Model fit verification.

Fit indices	Criteria	Model fit of research model	Pattern fitting
Chi-square (χ^2^)	The smaller the better	913.023	Pass
Degree of freedom (df)	The smaller the better	639	Pass
Normed Chi-square (χ^2^/df)	<3	1.429	Pass
RMSEA	<0.08	0.046	Pass
TLI (NNFI)	>0.9	0.948	Pass
CFI	>0.9	0.953	Pass
GFI	>0.8	0.821	Pass
AGFI	>0.8	0.792	Not pass (close to pass)
NFI	>0.8	0.861	Pass
IFI	>0.9	0.954	Pass

As is shown in Table 12, fit indices showed a better model fit, except for AGFI. AGFI was equal to 0.792, which is a little less than the standard value but close to 0.9. Therefore, its complexity remains to be improved and it is necessary to make a deep study in the future to define models. All in all, the structural equitation model is valid and matches the recycled data better, because most of the absolute indices meet the standard (GFI = 0.821, NFI = 0.861, IFI = 0.954, CFI = 0.953, TLI = 0.948).

#### Path Analysis

**FIGURE 5:**
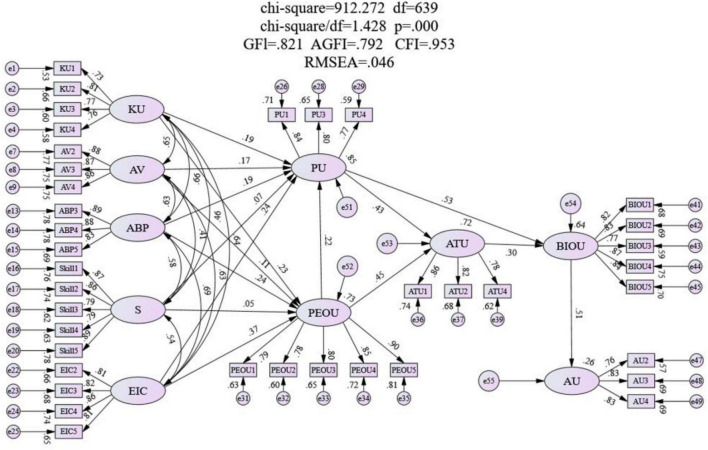
Integration of TAM with GLOs.

According to the path coefficient shown in Table 13, 85.3% of PU is related to KU, AV, ABP, S, EIC, and PEOU; 73.3% is related to KU, AV, ABP, S, and EIC; 63.9% of BIOU is related to ATU and PU; and 25.8% of AU is related to BIOU, which shows that the model is valid. Figure 5 shows the validity result of SEM.

**TABLE 13:** Regression coefficient.

Hypothesis		Relationship	Unstandardized coefficients		Standardized coefficients	*t*-Value	*P*-vaue	SMC
			Beta	SE	Beta			
H1	H1a	KU → PU	0.212	0.084	0.193	2.513	0.012*	0.853
	H1b	AV → PU	0.121	0.046	0.174	2.595	0.009**	
	H1c	ABP → PU	0.128	0.052	0.195	2.463	0.014**	
	H1d	S → PU	0.057	0.045	0.070	1.274	0.203	
	H1e	EIC → PU	0.227	0.079	0.237	2.883	0.004**	
H3		PEOU → PU	0.212	0.092	0.219	2.313	0.021*	
H2	H2a	KU → PEOU	0.255	0.091	0.226	2.800	0.005**	0.733
	H2b	AV → PEOU	0.081	0.052	0.114	1.575	0.115	
	H2c	ABP → PEOU	0.162	0.057	0.239	2.866	0.004**	
	H2d	S → PEOU	0.040	0.050	0.047	0.794	0.427	
	H2e	EIC → PEOU	0.371	0.081	0.375	4.552	***	
H4		PEOU → ATU	0.443	0.119	0.445	3.711	***	0.721
H5		PU → ATU	0.452	0.124	0.440	3.656	***	
H6		ATU → BIOU	0.292	0.108	0.308	2.694	***	0.639
H8		PU → BIOU	0.513	0.113	0.528	4.530	***	
H7		BIOU → AU	0.497	0.079	0.508	6.250	***	0.258

KU, knowledge and understanding; AV, attitudes and values; ABP, activity, behavior, and progression; S, skills; EIC, enjoyment, inspiration, and creativity; PEOU, perceived ease of use; PU, perceived usefulness; BIOU, behavioral intention to use; ATU, attitude toward using; AU, actual use. *Significant at p < 0.05, **significant at p < 0.01, ***significant at p < 0.001.

In this study, GLOs and TAM are combined and the result shows that KU, AU, ABP, and EIC significantly affect EIC. Besides, KU, ABP, and EIC significantly affect PEOU. PEOU significantly affects PU and ATU. PU significantly affects ATU. ATU significantly affects PU and BIOU. BIOU significantly affects AU. The model structure is shown in Figure 5.

Enjoyment, inspiration, and creativity play an important role in the experience. In the non-formal learning environment in the form of ICH tours, visitors are usually affected by emotions (Falk et al., 2012). New technology expands people’s imagination and creativity. With technology, people can create a joyful and practical learning environment in tourist attractions where PU, PEOU, ATU, and BIOU will be affected, improving AU, and making them learn some culture.

## Discussion

In this study, AR was applied to lantern exhibitions at Taiwan Lantern Festival to explore the characteristics of ICH tours in the era of the experience economy. Besides, questionnaires based on GLOs, TAM, accompanied by data collected were used. The result is shown below.

First, external variables impact PU and PEOU (Davis, 1989). With regard to external variables, KU, AV, ABP, and EIC have a significantly positive effect on PU and PEOU. It shows that the focus should be on KU, AV, ABP, and EIC when creating a new experience with AR. With its interaction, AR has become the best medium to convey information, thus having a great advantage in exhibition design. EIC has the most significant effect on PEOU, which is the same as the previous study results (Al-Aulamie et al., 2012; Kim and Park, 2019; Tao et al., 2019). Echoing its features, AR inspires visitors’ interest in its use and brings EIC, thus improving cultural awareness (Cranmer, 2019) and affecting future behavior (tom Dieck et al., 2018). Therefore, it is suggested that users should enjoy a vivid and interesting experience for ICH tours when digital technology is used to design the tour, improving their cultural awareness of ICH. Besides, study results show that skills will not affect PU and PEOU. This is because the description of skill dimensions is vague in GLOs, and it is hard to tell that they are technical learning, skill learning in knowledge, or skill learning with technology products. Thus, it is suggested that skills should be deleted in future studies where GLOs are used to measure ICH learning.

Second, with respect to TAM construction, results show that PEOU has a significantly positive effect on PU and ATU, which is corresponding with the original theoretical foundations of TAM (Davis, 1989). When visitors consider the AR system useful, their usefulness and attitude will undoubtedly increase. According to these results, PU has a significantly positive effect on ATU and BIOU, which is corresponding with previous studies (Davis, 1989; Esteban-Millat et al., 2018; Fan, 2020). When visitors consider the AR system useful, their attitude and behavioral intention will be better, which is corresponding with previous studies (Davis, 1989; Chung et al., 2015; Abdullah et al., 2016; Cheng and Yuen, 2018; Salloum et al., 2019). In addition, when visitors have a positive attitude toward the AR system, their BIOU AR will increase, thus improving visitors’ intention to use the AR system.

Third, with regard to AR technology, visitors found it hard to recognize some images with mobile devices, which is caused by the light of the environment and works displayed. González Vargas et al. (2020) pointed out that AR applications may be affected by lighting conditions. This is because the Lantern Festival is a kind of outdoor activity and will be affected by light on site. Besides, lanterns displayed themselves are illuminants, making it harder to recognize the images. In the future, importance should be attached to enhancing lanterns’ visibility to create more different visual effects.

Fourth, as is shown in the data, most visitors (73%) have surprisingly never gone to lantern exhibitions during the Lantern Festival. The Lantern Festival is a Chinese traditional activity, during which lantern exhibitions will be held in various cities. Dou et al. (2018) said that most of the public is not interested in ICH. However, when traditional lanterns are combined with AR technology, the exhibition successfully attracts a lot of visitors. It demonstrates that new media technology can improve visitors’ motivation to participate in ICH activities (Lo et al., 2019; Moorhouse et al., 2019) and give them a meaningful learning experience (Hsu and Liang, 2017; González Vargas et al., 2020), raising their cultural awareness (Lo et al., 2019).

Finally, many visitors indicated in their experience feedback that AR technology enhances visitors’ interaction with traditional lanterns. When a 2D image is shown on the mobile phone screen, visitors’ curiosity is inspired (Moorhouse et al., 2019), so they will keep using this system. As is said by V1, it was interesting to combine traditional lanterns with AR technology, making him want to complete all tasks. Additionally, it inspired visitors to change their behaviors toward ICH culture learning (Leue et al., 2015; Huang, 2019; Lo et al., 2019). It even inspired V2, V25, and V80 to know more about how to make lanterns. More importantly, the AR system inspired visitors’ positive experience behavior (Moorhouse et al., 2019; González Vargas et al., 2020). P105, P112, and P185 said, they had never seen the combination of AR technology with traditional lantern exhibitions, and wanted to experience and know more about it. Besides, V32 took part in it with their children, saying that the combination of AR and ICH tourism was a way for the public to know more about culture and learn about cultures with their children.

## Conclusion

This study contributes to the application of AR to the ICH tourism experience and the integration verification for GLOs and TAM. Results show that external variables, including KU, AV, ABP, and EIC, have a significant effect on PU and PEOU, and ATU and PU directly decide the intention to use AR. Finally, BIOU determines visitors’ actual usage.

This study explores the relationship between new technology and learning experience more completely than previous studies. The model developed in this study is useful for future reference in the application of digital technology to the tourism experience. In this study, learning is not limited to specific activity or device, thus it provides a user experience regardless of studying the impact of learning outcomes or tourism experience.

As a protector and transmitter, the public plays an important role in promoting sustainable ICH (Yan and Chiou, 2021). Cultural tourism will affect tourists’ cultural awareness and learning (Ismagilova et al., 2015). Therefore, it is a must to attract the public. As the gene of our national culture, ICH is particularly important for the development of human culture, social economy and civilization, and personal and national identity.

## Research Limitations and Future Work

This study is limited in several aspects. In terms of experimental samples, its size is small with only 200 visitors. This is because the number of visitors attending this activity is low during COVID-19 pandemic and interview time is short due to this kind of night activity. Therefore, this study may not apply to all ICH visitor experiences. As a result, future studies should expand their sample size for a more comprehensive study to confirm the generalization of the model. What is more, visitors’ experience is measured quantitatively and subjectively, and lack of objective data, for which it is suggested to verify these results quantitatively in future studies. Besides, this study is limited to the relation between ICH tours and technical systems in specific areas, but its results can be applied to more ICH tours, increasing samples to make a more comprehensive study.

In the cultural tour, visitors’ active involvement determines the validity of information communication and the quality of travel. Different people have different beliefs about involvement in exhibitions (Bastiaansen et al., 2019), such as young people paying attention to personalization and fashion; locals and foreigners have different needs for the same culture. In the era of the experience economy, visitors pursue more personalized experiences. With technology, festival events provide visitors with new experiences through creative transformation, creating connections with them and understanding them with empathy. Therefore, further study should be conducted later.

## Data Availability Statement

The original contributions presented in this study are included in the article/supplementary material, further inquiries can be directed to the corresponding author.

## Ethics Statement

Ethical review and approval was not required for the study on human participants in accordance with the local legislation and institutional requirements. The patients/participants provided their written informed consent to participate in this study.

## Author Contributions

X-ZL and XK contributed to the ideas of ICH research, collection of data, and empirical analysis. X-ZL, XK, and JK contributed to the data analysis, design of research methods, and tables. C-CC and X-ZL participated in developing a research design, writing, and interpreting the analysis. All authors contributed to the literature review and conclusion.

## Conflict of Interest

JK was employed by Beijing United Gas Engineering & Technology Co., Ltd. The remaining authors declare that the research was conducted in the absence of any commercial or financial relationships that could be construed as a potential conflict of interest.

## Publisher’s Note

All claims expressed in this article are solely those of the authors and do not necessarily represent those of their affiliated organizations, or those of the publisher, the editors and the reviewers. Any product that may be evaluated in this article, or claim that may be made by its manufacturer, is not guaranteed or endorsed by the publisher.
